# Left ventricular vorticity is marker of ventricular interdependency in pulmonary arterial hypertension

**DOI:** 10.1186/1532-429X-17-S1-P14

**Published:** 2015-02-03

**Authors:** Brett Fenster, Christopher A Podgorski, Joyce D Schroeder, Bryan Lin, Slade D Reisner, James Browning, Jean R Hertzberg, Kern J Buckner, Michal Schafer

**Affiliations:** 1 Division of Cardiology, National Jewish Health, Denver, CO, USA; 2 Bioengineering, University of Colorado Denver, Aurora, CO, USA; 3 Mechanical Engineering, University of Colorado Boulder, Boulder, CO, USA; 4 Radiology, National Jewish Health, Denver, CO, USA

## Background

Chronic right ventricular (RV) pressure and volume overload in pulmonary arterial hypertension (PAH) results in leftward shift of the interventricular septum and impaired left ventricular (LV) diastolic function. However, the impact of PAH-mediated interdependency on LV fluid mechanics and fluid/structure interactions in LV diastolic dysfunction is incompletely understood. 4D flow CMR analysis of LV inflow has demonstrated vortical formations during early (E wave) and late (A wave) filling. Vorticity is a novel hemodynamic parameter describing the local spinning nature of the fluid elements that measures the rotation of these vortices and may represent a novel way to assess the impact of interdependency on LV diastolic flow and function. Using LV systolic eccentricity index (EI) and diastolic tissue Doppler measurements as markers of ventricular interdependency, we aimed to determine if LV vorticity correlated with indices of interdependency in PAH subjects and controls.

## Methods

Thirteen PAH subjects (10 females/3 males) with PAH and 10 age-matched controls (7 females/3 males) underwent same-day 4D flow CMR and echocardiography. Mitral valve (MV) E and A velocities as well as MV septal and lateral early (e') and late (a') tissue Doppler velocities were measured. 4D flow CMR was performed with interleaved 3-directional velocity encoding (spatial resolution=3.5×2.6×3.0 mm3, α=15°, TE/TR=2.85/48.56 ms, venc=100cm/s, temporal resolution=50 ms) on a 1.5 T MRI system (Avanto, Siemens, Germany) using ECG gating and respiratory navigation. Vorticity integrated over the LV volume was calculated for all subjects at peak E and A wave filling using noise-corrected data (Paraview, Kitware, Clifton, NY). LV EI was computed using 2D CMR short axis images. Intervariable correlations were performed using Spearman rho coefficients (JMP 10.0, Cary, NC). 2-tailed Students T-Tests were performed for intergroup comparisons and reported as means ± 1 standard deviation.

## Results

LV E wave vorticity was significantly decreased in the PAH group compared to controls (1952 ± 1599 vs. 3636 ± 1021 s^-1^, p=0.006). Conversely, there was no difference in LV A wave vorticity (2687±1519 vs. 1754±1864, p=0.21). LV E vorticity significantly correlated with MV E (Rho 0.63, p=0.002), E/A (Rho 0.51, p=0.014), septal e' (Rho=0.58, p=0.004) and lateral e' (Rho=0.59, p=0.003) velocities. In addition, LV A vorticity correlated with MV A (Rho 0.51, p=0.014), septal a' (Rho 0.46, 0.027) and lateral a' (rho=0.60, p=0.003) velocities. Both LV E and A vorticity both showed significant correlations with LV eccentricity index (rho=-0.47, p=0.023 and rho=0.42, p=0.042, respectively).

## Conclusions

LV vorticity correlates with multiple noninvasive markers of ventricular interdependency including LV EI and diastolic parameters in a cohort of PAH subjects and controls. 4D CMR-derived vorticity may represent a novel research tool to the study the role of fluid-tissue biomechanical interactions in LV-RV interdependency.

## Funding

This study was funded through a grant from Siemens Healthcare.

